# The prognostic value of ASPECTS in specific regions following mechanical thrombectomy in patients with acute ischemic stroke from large-vessel occlusion

**DOI:** 10.3389/fneur.2024.1372778

**Published:** 2024-04-12

**Authors:** Yu Zou, Jianglong Tu, Pengxin Hu, Xihai Zhao, Xiaoping Tang

**Affiliations:** ^1^Department of Radiology, The Second Affiliated Hospital, Jiangxi Medical College, Nanchang University, Nanchang, China; ^2^Department of Neurology, The Second Affiliated Hospital, Jiangxi Medical College, Nanchang University, Nanchang, China; ^3^Center for Biomedical Imaging Research, Department of Biomedical Engineering, School of Medicine, Tsinghua University, Beijing, China; ^4^School of Medicine, National Graduate College for Engineers, Tsinghua University, Beijing, China

**Keywords:** core infarct site, core infarct volume, the Alberta stroke program early CT score, artificial intelligence, mechanical thrombectomy, acute ischemic stroke

## Abstract

**Objective:**

The aim of this study is to investigate the relationship between the volume of specific regional infarction and the prognosis of patients who undergo mechanical thrombectomy (MT) for acute large vessel occlusion.

**Methods:**

In this study, we collected the clinical and imaging features of patients with unilateral acute anterior circulation ischemic stroke from January 2021 to June 2023 in the Second Affiliated Hospital of Nanchang University. All patients underwent CT perfusion and non-contrast CT scan before MT. The ASPECTS was assessed based on imaging data, and artificial intelligence was used to obtain the percentage of infarction in each of the 10 regions of ASPECTS. According to the modified Rankin Scale, the patients were divided into the good prognosis group and poor prognosis group at the 90-day follow-up. Various indicators in the two groups were compared. Multivariable logistic regression was used to assess the risk factors for poor prognosis. The relationship between core infarction volume and the probability of poor prognosis was plotted to analyze the trend of poor prognosis with changes in the proportion of infarction volume. Finally, a receiver operating characteristic curve was constructed to analyze the predictive ability on poor prognosis.

**Results:**

A total of 91 patients were included, with 58 patients having a good prognosis (mRS ≤ 2) and 33 patients having a poor prognosis (mRS ≥ 3). Multivariate analysis showed that NIHSS score and core infarction involving the internal capsule and M6 region were independent risk factors for poor prognosis. According to the linear correlation, a higher ratio of core infarction volume in the internal capsule or M6 region was linked to an increased risk of a poor prognosis. However, the non-linear analysis revealed that the prognostic impact of core infarction volume was significant when the ratio was greater than 69.7%. The ROC curve indicated that the combination of NIHSS score, infarct location, and the ratio of infarct volume has an AUC of 0.87, with a sensitivity of 84.8% and a specificity of 84.5%.

**Conclusion:**

It is important to examine the location and volume of the infarct in the internal capsule and M6 when deciding whether to do a MT.

## 1 Introduction

Currently, mechanical thrombectomy (MT) is one of the main methods used to recanalize occluded blood vessels in acute ischemic stroke (AIS). However, studies have shown that not all early stroke patients benefit from this treatment, and blind treatment may increase the risk of intracranial hemorrhage (1, 2). Thus, it is critical to accurately evaluate early ischemia alterations. Clinicians typically use the National Institutes of Health Stroke Scale (NIHSS) to assess the degree of neurological deficit in acute stroke patients. However, this scale, which contains many items, does not quantify clinical symptoms accurately. It relies mainly on the subjective judgment of the scorer, which has certain limitations (3, 4).

Barber et al. (5) proposed the Alberta Stroke Program Early CT Score (ASPECTS) method. However, the manual ASPECTS method relies on the scorer’s experience and lacks consistency. This can result in delayed and inaccurate diagnosis during the early stages of the disease. Fortunately, with advancements in medical engineering and the application of artificial intelligence, CT and MRI imaging data in stroke patients can now be processed more effectively (6). This eliminates the impact of subjective factors and enhances the accuracy and consistency of diagnosis. Previous studies have shown that patients with ASPECTS ≥6 are more likely to benefit from endovascular treatment (7–10). However, recent research has found that some AIS patients with lower ASPECTS can also achieve good outcomes after successful recanalization with MT (11–13). This suggests that the ASPECTS has certain limitations, and poor prognosis may be related to the location of the infarction. Equating infarction locations may prematurely exclude patients who could benefit from MT in the early stages. Therefore, the aim of this study is to explore the relationship between the 10 regions and infarct volume assessed by ASPECTS and the prognosis of patients after MT for acute large vessel occlusion.

## 2 Materials and methods

### 2.1 General information

Clinical and imaging data of patients diagnosed with acute anterior circulation large vessel occlusive ischemic stroke at the Second Affiliated Hospital of Nanchang University from January 2021 to June 2023 were collected. Inclusion criteria were as follows: (1) Age ≥ 18 years; (2) Patients with unilateral anterior circulation large vessel occlusion AIS; (3) Mechanical thrombectomy performed within 24 h of symptom onset; (4) Preoperative NCCT and CTP examinations were conducted. Exclusion criteria were as follows: (1) Bilateral arterial occlusion or posterior circulation AIS; (2) Known intracranial hemorrhage, trauma, or tumor; (3) Incomplete clinical and imaging data; (4) Poor image quality. The research protocol was approved by the Ethics Committee of the Second Affiliated Hospital of Nanchang University.

### 2.2 Instrument

A GE 256-slice spiral CT scanner was used. Patients were instructed to keep their heads stable. First, a whole-brain NCCT scan was performed to exclude intracranial hemorrhage. The scanning range extended from the outer canthus of the eye to the vertex, with the following parameters: scanning voltage of 120 kV, scanning current of 300 mA, slice thickness of 5 mm, and interslice spacing of 5 mm. Subsequently, a CTP examination was conducted. The dose was 1.5 kg/mL of iodinated contrast agent (370 mgI/mL) was injected via the cubital vein at a rate of 5.0 mL/s. The scan was initiated 5 s after the injection, and the total scan time was at least 50 s, with an interval of less than 4 s between two scans. The scanning parameters for CTP were as follows: scanning voltage of 80 kV, scanning current of 150 mAs, and slice thickness of 5 mm.

### 2.3 Imaging assessment

**Figure 1:**
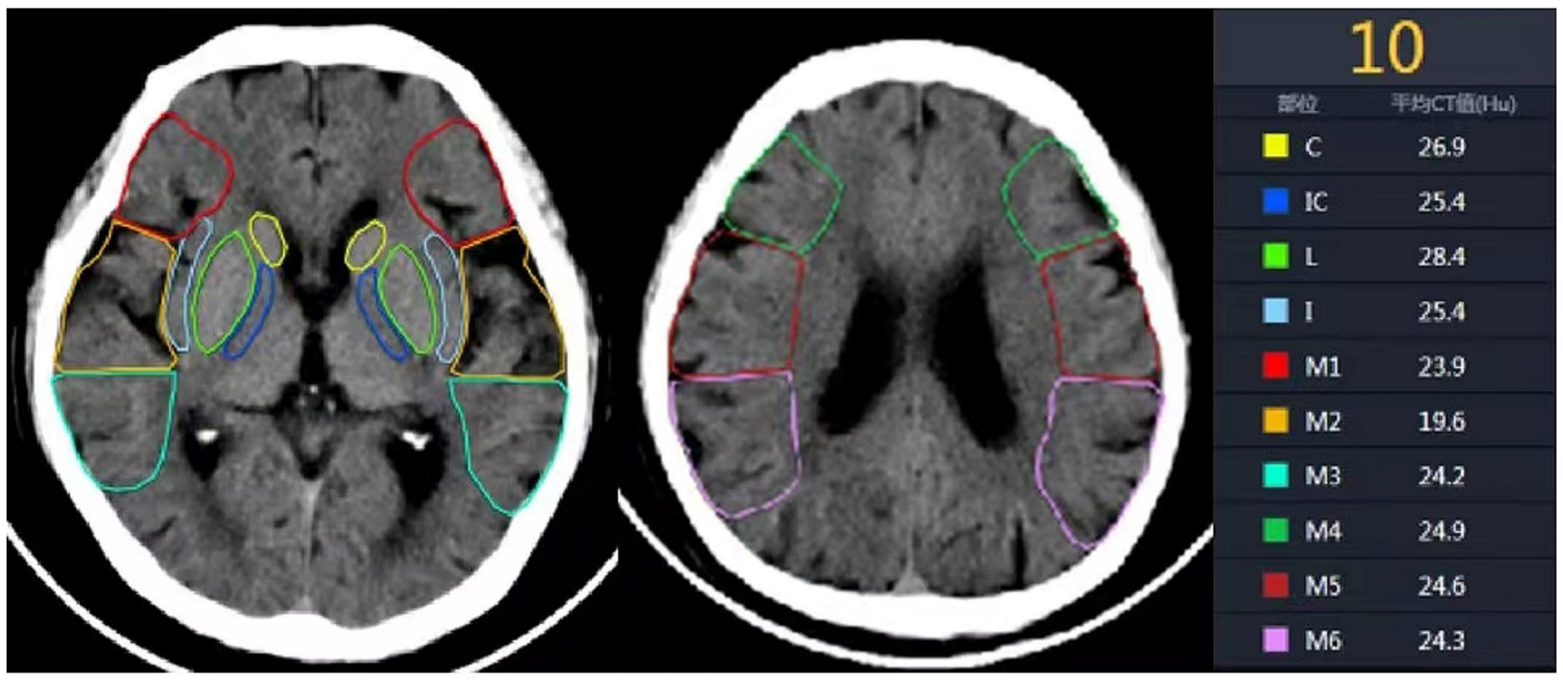
Ten anatomical regions of ASPECTS. C, Caudate nucleus; IC, Internal capsule; L, Lentiform nucleus; I, Insula; M1, anterior inferior frontal lobe; M2, temporal lobe; M3, inferior parietal and posterior temporal lobe; M4, anterior superior frontal lobe; M5, precentral and superior frontal lobe; M6, superior parietal lobe.

All preoperative NCCT and CTP raw data of the patients were processed using F-stroke software (NeuroBlem Ltd. Shanghai, version: V 1.0.26). In the software, the core infarct location refers to the region with relative cerebral blood flow less than 30%, and the core infarct proportion refers to the percentage of the core infarct volume in the corresponding anatomical area relative to the total volume of that area (14). Additionally, two senior radiologists with intermediate or higher professional titles combined with artificial intelligence analysis to assess the imaging data and calculate the ASPECTS (Figure 1).

### 2.4 Clinical data and follow-up

The general information of all enrolled patients was recorded, including age, gender, history of hypertension, history of hyperglycemia, history of hyperlipidemia, history of elevated homocysteine, history of coronary heart disease, history of atrial fibrillation, smoking history, bridging therapy, baseline NIHSS score, onset to door time (ODT), door to puncture time (DPT), onset to puncture time (OPT), and puncture to recanalization time (PRT). After a 90-day follow-up, the patients were divided into the good prognosis group (mRS ≤ 2) and the poor prognosis group (mRS ≥ 3) according to the modified Rankin Scale (mRS).

### 2.5 Statistical analysis

Statistical analysis was performed using SPSS 27.0 software. The Shapiro–Wilk test was used to assess the normality of quantitative data. Normally distributed continuous variables were presented as mean ± standard deviation (x ± s), and the independent samples t-test was used for comparisons between the two groups. Non-normally distributed continuous variables were presented as median (Q1, Q3), and the Mann–Whitney U test was used for comparisons between the two groups. Categorical variables were presented as frequency (%), and the χ^2^ test or Fisher’s exact test was used for comparisons between the two groups. Multivariate logistic regression analysis was then conducted to identify independent risk factors for poor prognosis.

The relationship between the percentage of core infarction volume and the probability of poor prognosis, as well as the odds ratio (OR), was assessed. Finally, receiver operating characteristic (ROC) curves were constructed to evaluate the predictive ability of the percentage of core infarction volume and other indicators for adverse prognosis by comparing the area under the curve (AUC). A significance level of *p* < 0.05 was considered statistically significant.

## 3 Results

### 3.1 Baseline characteristics

**Figure 2:**
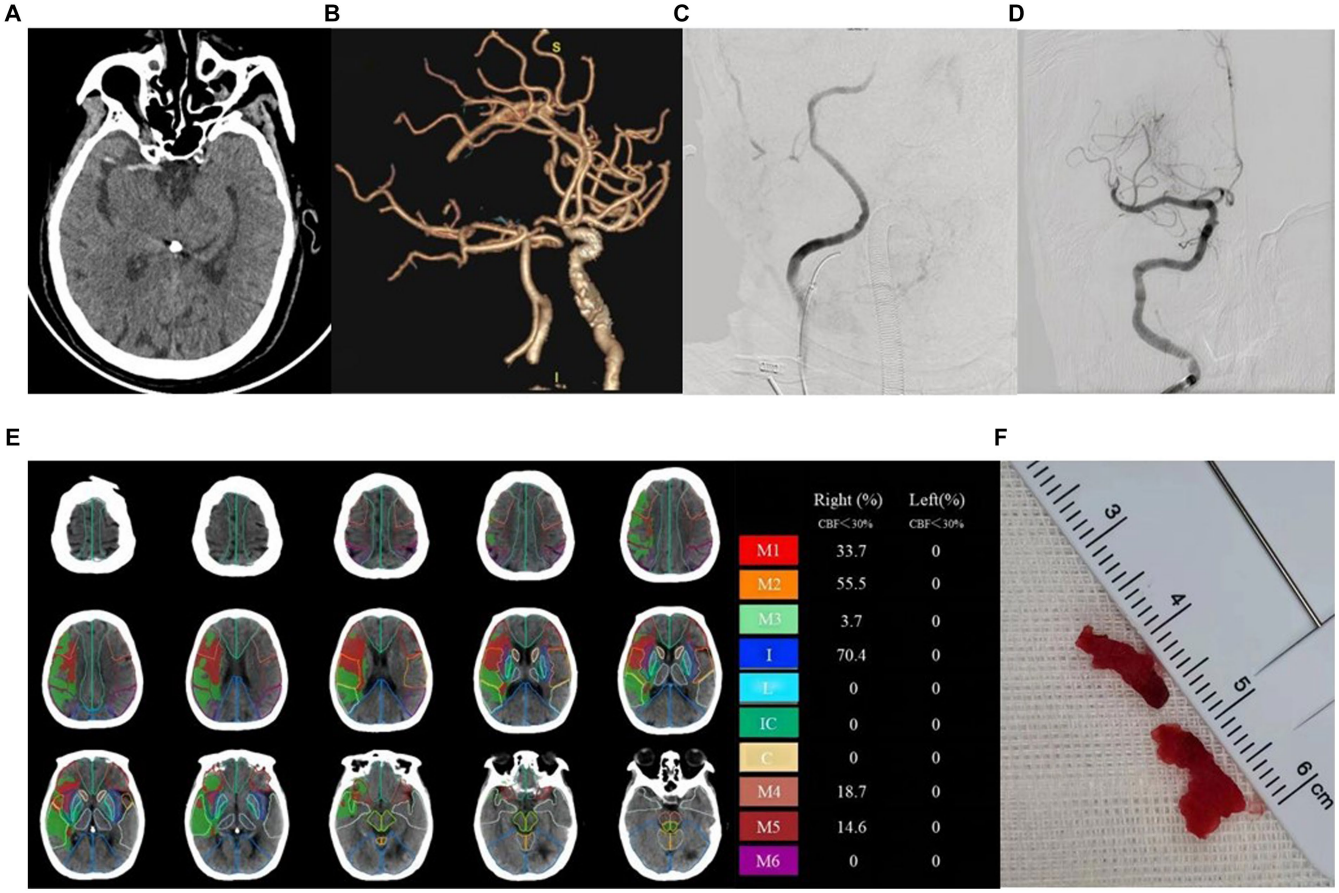
Example of patient with good prognosis (mRS≤2) and low ASPECT scores. Female, 78 years old, with limb weakness 1 day. ASPECTS is 4 points. **(A)** NCCT shows dense middle cerebral artery sign on the right side. **(B)** CTA MIP shows occlusion of the right internal carotid artery. **(C)** DSA shows stenosis of the right internal carotid artery. **(D)** DSA shows recanalization of the right internal carotid artery after surgery. **(E)** The core infarction involves the M1, M2, M3, internal capsule, M4, and M5 region, along with their respective infarction percentages. **(F)** Operation sample.

**Figure 3:**
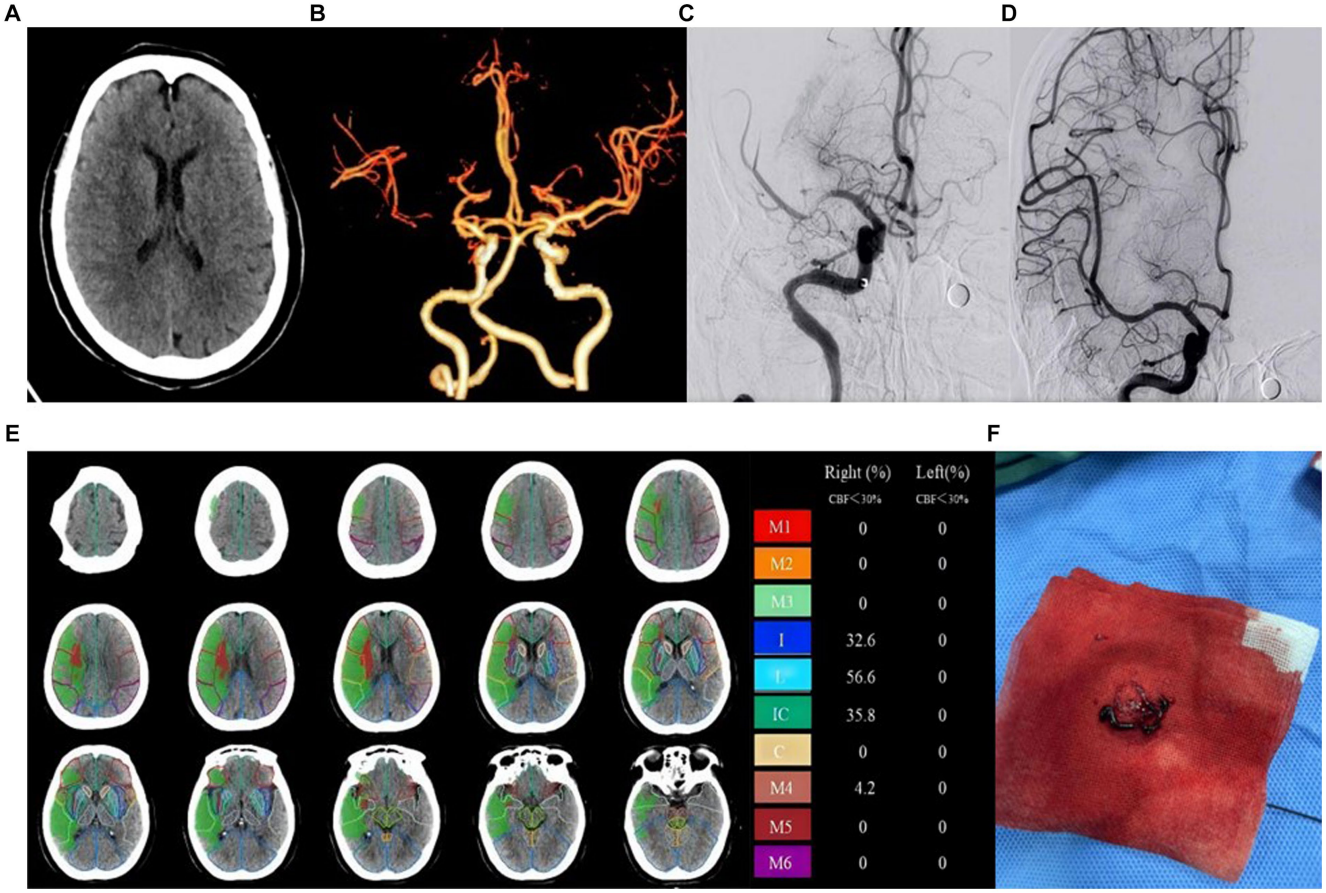
Example of patient with poor prognosis (mRS > 2) and high ASPECT scores. Female, 73 years old, woke up with limb weakness for 6 hours. ASPECTS is 6 points. **(A)** NCCT shows patchy hypodensity in the right cerebral hemisphere. **(B)** CTA MIP shows occlusion of the right middle cerebral artery. **(C)** DSA shows occlusion of the right middle cerebral artery. **(D)** DSA shows recanalization of the right middle cerebral artery. **(E)** The core infarction involves the insula, lentiform nucleus, internal capsule, and M4, along with their respective infarction percentages. **(F)** Operation sample.

A total of 91 patients were included in the study, with 58 cases in the good prognosis group (44 males and 14 females; Figure 2) and 33 cases in the poor prognosis group (21 males and 12 females; Figure 3). The PRT was longer in the poor prognosis group compared to the good prognosis group [41.5(35, 53.8) vs. 52 (42.5,90)]. The baseline NIHSS scores were higher in the poor prognosis group compared to the favorable prognosis group [14 (7,20) vs. 8(6,11), *p* = 0.01]. The ASPECTS were lower in the poor prognosis group compared to the favorable prognosis group [5(3,8) vs. 8(6,9), *p* = 0.01]. There were no statistically significant differences between the two groups in other indicators (*P* > 0.05; Table 1).

**Table 1:** Comparison of baseline data between the good prognosis group and poor prognosis group.

	Good prognosis group (*n* = 58)	Poor prognosis group (*n* = 33)	*P-*value
Age (year, xˉ ± s)	61 ± 11	65 ± 12	0.075^a^
Male [*n* (%)]	44 (75.9)	21 (63.6)	0.215^b^
ODT [min, M (Q1, Q3)]	321.5 (152.3,566.3)	300 (194,790)	0.506^c^
DPT [min, M (Q1, Q3)]	216 (165,277.5)	185 (135,315.5)	0.460^c^
OPT [min, M (Q1, Q3)]	533 (371.5,851.3)	535 (429,925)	0.435^c^
PRT [min, M (Q1, Q3)]	41.5 (35,53.8)	52 (42.5,90)	0.019^c^
Bridging therapy [*n* (%)]	8 (13.8)	7 (21.2)	0.359^b^
Hypertension [*n* (%)]	40 (67.0)	24 (72.7)	0.706^b^
Hypercholesterolemia [*n* (%)]	12 (20.7)	8 (24.2)	0.694^b^
Hyperlipidemia [*n* (%)]	13 (22.4)	6 (18.2)	0.633^b^
Elevated homocysteine [*n* (%)]	29 (50.0)	14 (42.4)	0.486^b^
Smoking [*n* (%)]	11 (19.0)	6 (18.2)	0.927^b^
Drinking [*n* (%)]	8 (13.8)	5 (15.2)	0.859^b^
Atrial fibrillation [*n* (%)]	13 (22.4)	12 (36.4)	0.152^b^
Coronary heart disease [*n* (%)]	4 (6.9)	3 (9.1)	0.706^b^
Prior stroke [*n* (%)]	10 (17.2)	6 (18.2)	0.910^b^
Baseline NIHSS score [M (Q1, Q3)]	8 (6,11)	14 (7,20)	0.010^c^
ASPECTS [M (Q1, Q3)]	8 (6,9)	5 (3,8)	0.010^c^

ODT, onset to door time; DPT, door to puncture time; OPT, onset to puncture time; PRT, puncture to recanalization time; NIHSS, National Institutes of Health Stroke Scale; ASPECTS, the Alberta Stroke Program Early CT Score. ^a^Shapiro–Wilk test. ^b^χ^2^ test. ^c^Wilcoxon Mann–Whitney *U*-test.

### 3.2 Relationship between core infarct involvement and prognosis

**Figure 4:**
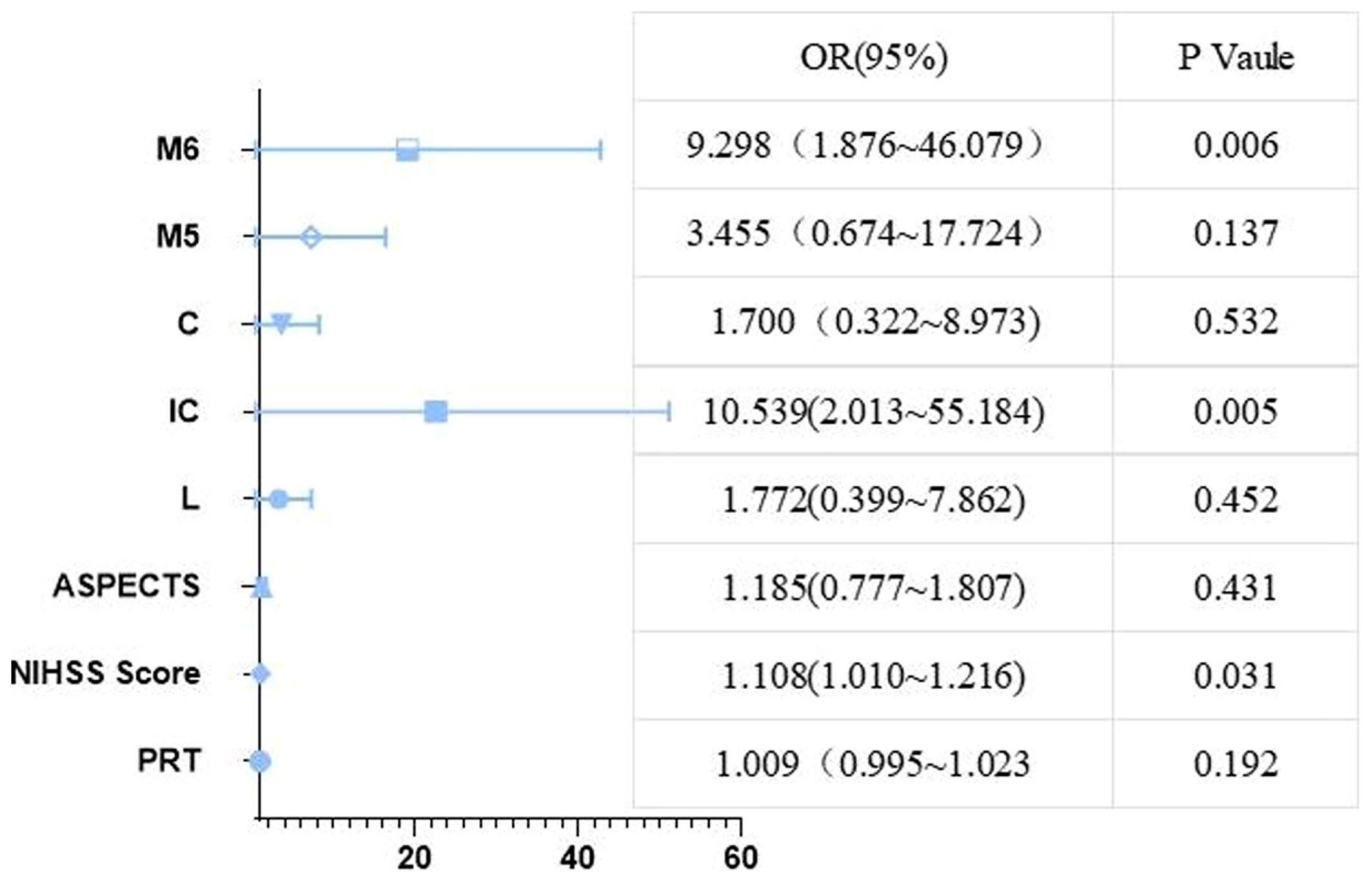
A forest plot of the multivariate regression analysis that influences poor prognosis.

Firstly, the χ^2^ test was performed to assess the relationship between core infarction in M1, M2, M3, M4, M5, M6, insula, lentiform nucleus, internal capsule and caudate nucleus and prognosis. Significant differences were found between the two groups with regard to core infarcts in the lentiform nucleus, internal capsule, caudate nucleus, M5 and M6 (Table 2). The factors that showed significant differences in the above tests, along with ASPECTS and NIHSS scores, were included as independent variables in a multiple Logistic regression model. The results, as shown in Table 3 and Figure 4, indicated that infarction in the internal capsule (OR = 10.54, *p* = 0.005) and M6 region (OR = 9.3, *p* = 0.006) were independent predictors of poor prognosis.

**Table 2:** Comparison of imaging features between the good prognosis group and the poor prognosis group.

	Good prognosis group (*n* = 58)	Poor prognosis group (*n* = 33)	*P*
**Core infarction site [*n* (%)]**			
M1	19 (32.8)	17 (51.5)	0.079^b^
M2	25 (43.1)	16 (48.5)	0.62^b^
M3	15 (25.9)	13 (39.4)	0.179^b^
I	24 (41.4)	19 (57.6)	0.137^b^
L	18 (31.0)	18 (54.5)	0.027^b^
IC	6 (10.3)	16 (48.5)	<0.001^b^
C	6 (10.3)	10 (30.3)	0.016^b^
M4	21 (36.2)	16 (48.5)	0.252^b^
M5	20 (34.5)	19 (57.6)	0.032^b^
M6	6 (3.4)	14 (42.4)	<0.001^b^

I, Insula; L, Lentiform nucleus; IC, Internal capsule; C, Caudate nucleus; ^b^χ^2^test.

**Table 3:** Multivariate logistic regression models used to identify independent predictors of poor prognosis.

Variable	β	S.E	Waldχ^2^	OR	95%CI	*P*
PRT	0.009	0.007	1.70	1.01	1.00~1.02	0.192
Baseline NIHSS score	0.10	0.05	4.67	1.11	1.01~1.22	0.031
ASPECTS	0.17	0.22	0.62	1.19	0.78~1.81	0.431
L	0.57	0.76	0.57	1.77	0.40~7.86	0.452
IC	2.36	0.85	7.78	10.54	2.01~55.18	0.005
C	0.53	0.85	0.39	1.7	0.32~8.97	0.532
M5	1.24	0.83	2.21	3.46	0.67~17.72	0.137
M6	2.23	0.82	7.46	9.3	1.88~46.08	0.006

Input method; PRT, puncture to recanalization time; NIHSS, National Institutes of Health Stroke Scale; ASPECTS, the Alberta Stroke Program Early CT Score; OR, Odds Ratio; L, Lentiform nucleus; IC, Internal capsule; C, Caudate nucleus.

### 3.3 The relationship between the proportion of core infarction in each region and prognosis will be examined

**Figure 5:**
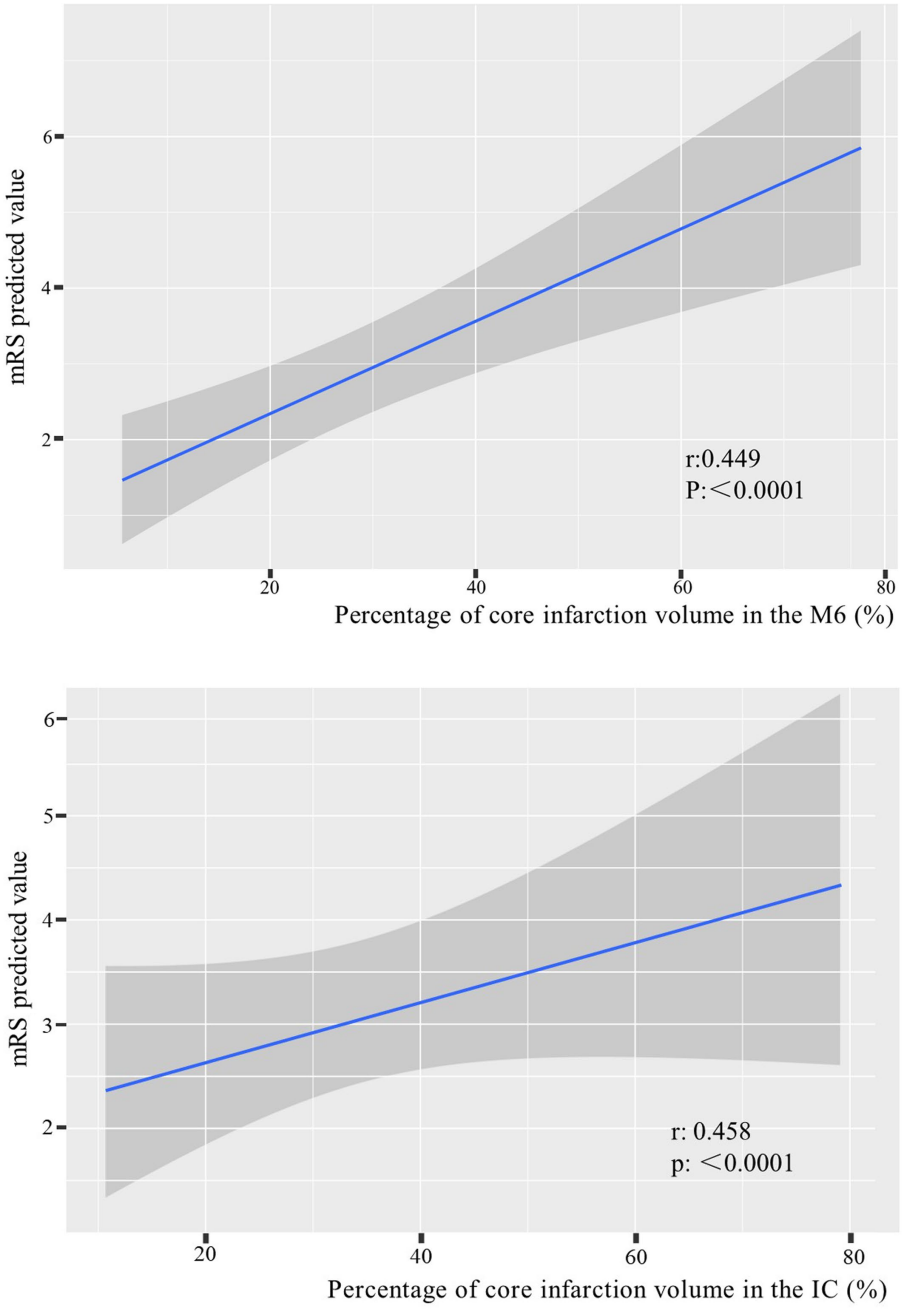
The relationship between the percentage of core infarction volume in the internal capsule or M6 and prognosis.

**Figure 6:**
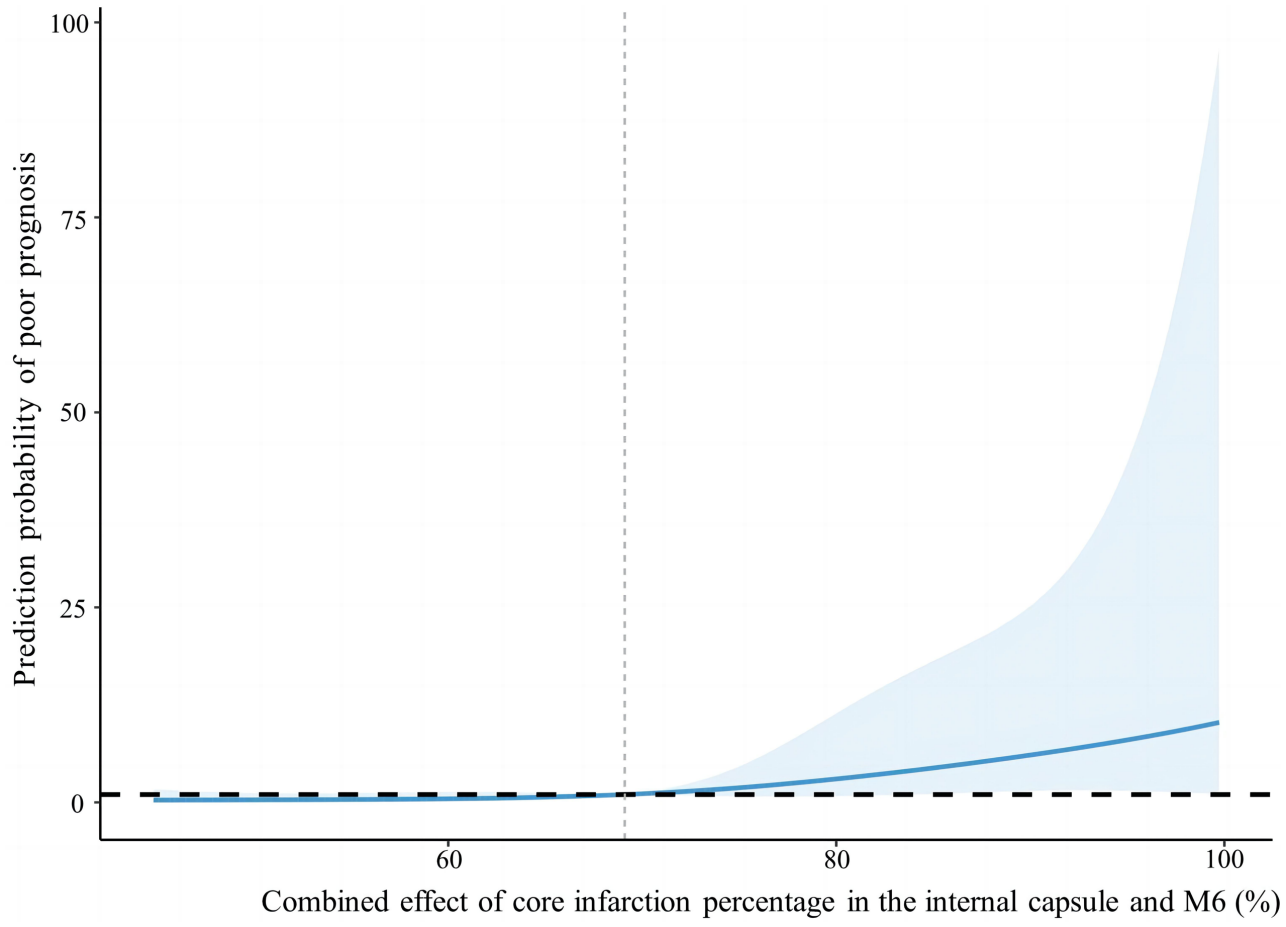
Nonlinear relationship between combined effect of core infarction percentage in the internal capsule and M6 and poor prognosis.

**Figure 7:**
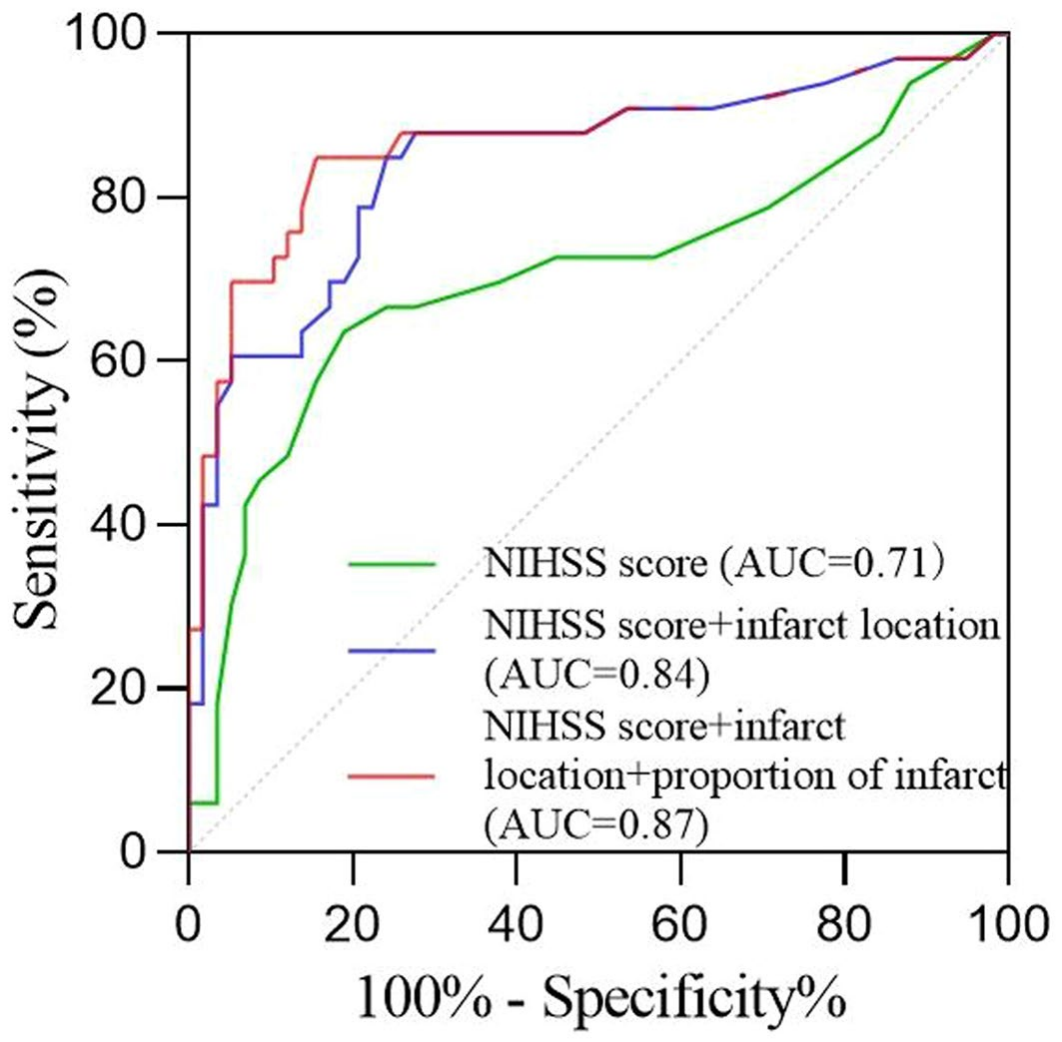
The receiver operating characteristic curve of the prognosis prediction of the NIHSS score and imaging feature.

According to the foregoing conclusion, a Wilcoxon test was performed on the proportion of core infarction in the internal capsule and M6 between the two groups and the results were statistically significant. Spearman’s rank correlation was used to analyze the relationship between the proportion of core infarction volume in internal capsule or M6 and prognosis. The results indicated that as the proportion of core infarction volume increased, the likelihood of a poor prognosis also increased (Figure 5). A restricted cubic spline was fitted with the number of nodes set as 4 to consider the possible non-linear relationship between the joint effect of the two regions and prognosis. The relationship curve between the proportion of core infarction volume and the probability of poor prognosis as well as OR was plotted. It can be observed that when the proportion of core infarction is relatively small (≤69.7%), it has a minimal impact on the occurrence of poor prognosis. However, once the proportion of core infarction volume exceeds 69.7%, its contribution significantly increases (Figure 6). Finally, models were constructed using baseline NIHSS score, baseline NIHSS score combined with the affected location, and baseline NIHSS score combined with the affected location and proportion of core infarction. The ROC curve showed that the combination of baseline NIHSS score with the affected location and proportion of core infarction had the best predictive effect for poor prognosis (Table 4; Figure 7).

**Table 4:** The value of NIHSS score, core infarct location, and infarct proportion in assessing the risk of poor prognosis in patients.

	AUC	95%CI	*P*	sensitivity	specificity
NIHSS score	0.71	0.58~0.83	<0.01	63.6	81.0
NIHSS score + infarct location	0.84	0.75~0.94	<0.01	84.8	75.9
NIHSS score + infarct location + proportion of infarct	0.87	0.78~0.96	<0.01	84.8	84.5

## 4 Discussion

The results of this study revealed that the PRT was lower in the group with good prognosis compared to the group with poor prognosis and ASPECTS in good prognosis group was higher than the last group. Additionally, the NIHSS score was identified as an independent risk factor for poor prognosis. Moreover, this study utilized artificial intelligence to determine the regions and proportions of core infarction. Additionally, it investigated the correlation between regional infarction and its size with the clinical prognosis in patients who underwent MT. The results revealed that infarction in the internal capsule and M6 region, along with the volume of infarction, were strongly linked to a poor prognosis. Furthermore, these factors were identified as independent risk factors for poor prognosis.

A study by Romain Bourcier et al. (15) confirmed a significant association between PRT and the prognosis of patients after MT. Lower PRT was found to be associated with better reperfusion status, which aligns with the results of this study. Previous studies have also demonstrated that baseline NIHSS score and ASPECTS are prognostic factors for patients undergoing mechanical thrombectomy for acute ischemic stroke (16–18), which is consistent with the findings of this study. The ASPECTS reflects the extent of core infarction, and a lower score indicates a larger infarction area and a higher likelihood of poor prognosis. The NIHSS score is a commonly used indicator to assess the severity of neurological deficits. A higher NIHSS score indicates a more severe neurological deficit and a more difficult recovery after surgery. In addition, previous literature (19–21) has shown that the OPT is closely related to poor prognosis. However, this factor was not statistically significant in this study, which may be due to increased awareness of stroke in recent years and the improvement of emergency green channels, as all patients included in this study had an OPT within 12 h.

The volume of core infarction and the volume of ischemic penumbra have been shown to be closely related to the postoperative clinical prognosis of patients with acute ischemic stroke. In recent years, studies have also shown a certain correlation between clinical prognosis and the location of infarction. A study conducted by Charlotte Rosso et al. (22) revealed that the preservation of the thalamus, internal capsule, and cortical M5 in left-sided AIS were independent predictors of a good prognosis. On the other hand, the cortical M3 and M6 were found to be associated with clinical prognosis in right-sided AIS. Additionally, Haranhalli et al. (23) suggested that infarction in the insula, M2, and M5 was linked to high mRS scores. Pietro Panni et al. (24) suggested that involvement of the M6 in right-sided stroke and involvement of the internal capsule in left-sided stroke were independent risk factors for poor prognosis. There are some discrepancies in the above studies. The reason for these discrepancies may be that the ASPECTS in those studies were based on subjective judgments, whereas in this study, all ASPECTS of NCCT and CTP were analyzed by physicians using software analysis to minimize errors.

A meta-analysis conducted by Seyedsaadat et al. (25) revealed that infarction in the M6 and M3 regions, as assessed by ASPECTS on NCCT images, was more predictive of adverse functional outcomes compared to other anatomical regions. This finding is consistent with the results of our study. The significant association between infarction in the internal capsule and poor prognosis may be attributed to the fact that the internal capsule serves as a convergence point for incoming and outgoing fibers from various structures, connecting the cerebral cortex, thalamus, brainstem, and spinal cord. As a result, patients with internal capsule infarction may experience severe neurological deficits. The M6 region includes the primary motor cortex, and even relatively small infarctions in this region can lead to symptoms related to motor impairment and aphasia (26).

We used artificial intelligence to obtain the percentage of core infarction volume to corresponding region volume in the 10 regions of the ASPECTS, quantifying the core infarction volume in each region and analyzing the impact of regional core infarction proportion on prognosis. The results showed that the core infarction volume in the internal capsule and M6 was also an independent predictor of poor prognosis and had significant predictive value for prognosis. The larger the proportion of core infarction volume in the internal capsule or M6, the more likely the patient was to have a high mRS score at 90 days. The combined effect of the internal capsule and M6 region on prognosis showed a non-linear relationship. When the proportion of core infarction volume was ≤69.7%, there was a weaker correlation with prognosis improvement. However, when the proportion of core infarction volume exceeded 69.7%, the correlation with prognosis significantly increased. Previous studies have primarily focused on the role of total core infarction volume in predicting prognosis. However, this study goes a step further by demonstrating the significance of regional infarction. Based on the ROC curve, the diagnostic performance was found to be highest when combining the location and volume of the ischemic core with the clinical NIHSS score. This information can assist clinicians in early identification of acute stroke patients who may benefit from thrombectomy, ultimately leading to more accurate and appropriate treatment for a larger number of patients.

This study addressed two inherent limitations of the ASPECTS using artificial intelligence. First, it assumed that the impact of infarction in each region on prognosis is equal. Second, it assumed that regardless of the size of infarction volume within a region, any infarction in that region has the same impact on prognosis.

Furthermore, this study has limitations. Although this study thoroughly discussed the relationship between ASPECTS anatomical region infarction and prognosis, it did not separately investigate the relationship between the involved regions and their proportions in left and right hemisphere AIS patients and the prognosis after MT. Additionally, this study did not include infarctions in the posterior circulation region. Finally, this study is a retrospective small-sample study conducted at a single center, and further research with larger sample sizes and multi-center studies are needed for validation.

In conclusion, this study further demonstrated the relationship between infarction in the internal capsule and M6 region and their volumes with the prognosis of patients after MT. Therefore, clinicians should consider the infarction status in these regions when deciding whether to perform thrombectomy, which can help assess the prognosis of patients.

## Data availability statement

The raw data supporting the conclusions of this article will be made available by the authors, without undue reservation.

## Ethics statement

The studies involving humans were approved by the Ethics Committee of the Second Affiliated Hospital of Nanchang University. The studies were conducted in accordance with the local legislation and institutional requirements. Written informed consent for participation was not required from the participants or the participants’ legal guardians/next of kin in accordance with the national legislation and institutional requirements.

## Author contributions

YZ: Data curation, Investigation, Methodology, Writing – original draft. JT: Software, Writing – review & editing. PH: Writing – original draft. XZ: Writing – review & editing. XT: Methodology, Supervision, Writing – review & editing.

## References

[1] Wassélius J Arnberg F Von Euler M Wester P Ullberg T. Endovascular thrombectomy for acute ischemic stroke. J Intern Med. (2022) 291:303–16. doi: 10.1111/joim.13425 35172028

[2] Zhang X Xie Y Wang H Yang D Jiang T Yuan K. Symptomatic intracranial hemorrhage after mechanical thrombectomy in Chinese ischemic stroke patients. Stroke. (2020) 51:2690–6. doi: 10.1161/STROKEAHA.120.030173, PMID: 32811387

[3] Josephson SA Hills NK Johnston SC. NIH stroke scale reliability in ratings from a large sample of clinicians. Cerebrovasc Dis. (2006) 22:389–95. doi: 10.1159/000094857, PMID: 16888381

[4] Antipova D Eadie L Macaden A Wilson P. Diagnostic accuracy of clinical tools for assessment of acute stroke: a systematic review. BMC Emerg Med. (2019) 19:49. doi: 10.1186/s12873-019-0262-1, PMID: 31484499 PMC6727516

[5] Pexman JH Barber PA Hill MD. Use of the Alberta Stroke Program Early CT Score (ASPECTS) for assessing CT scans in patients with acute stroke. AJNR Am J Neuroradiol. (2001) 22:1534–1542. 11559501 PMC7974585

[6] Shafaat O Bernstock JD Shafaat A Yedavalli VS Elsayed G Gupta S. Leveraging artificial intelligence in ischemic stroke imaging. J Neuroradiol. (2022) 49:343–51. doi: 10.1016/j.neurad.2021.05.001, PMID: 33984377

[7] Avasarala J. Letter by avasarala regarding article, “2015 aha/asa focused update of the 2013 guidelines for the early management of patients with acute ischemic stroke regarding endovascular treatment: a guideline for healthcare professionals from the american heart association/american stroke association”. Stroke. (2015) 46:e234. doi: 10.1161/STROKEAHA.115.010716, PMID: 26443830

[8] Bonney PA Walcott BP Singh P Nguyen PL Sanossian N Mack WJ. The continued role and value of imaging for acute ischemic stroke. Neurosurgery. (2019) 85:S23–30. doi: 10.1093/neuros/nyz068, PMID: 31197337

[9] Phan K Saleh S Dmytriw AA Maingard J Barras C Hirsch JA. Influence of aspects and endovascular thrombectomy in acute ischemic stroke: a meta-analysis. J Neuro Intervention Surg. (2019) 11:664–9. doi: 10.1136/neurintsurg-2018-014250, PMID: 30415223

[10] Jadhav AP Desai SM Jovin TG. Indications for mechanical thrombectomy for acute ischemic stroke. Neurology. (2021) 97:S126–36. doi: 10.1212/WNL.0000000000012801 34785611

[11] Almallouhi E Al Kasab S Hubbard Z Bass EC Porto G Alawieh A. Outcomes of mechanical thrombectomy for patients with stroke presenting with low Alberta stroke program early computed tomography score in the early and extended window. JAMA Netw Open. (2021) 4:e2137708. doi: 10.1001/jamanetworkopen.2021.37708, PMID: 34878550 PMC8655598

[12] Manceau P-F Soize S Gawlitza M Fabre G Bakchine S Durot C. Is there a benefit of mechanical thrombectomy in patients with large stroke (dwi-aspects ≤ 5)? Eur J Neurol. (2018) 25:105–10. doi: 10.1111/ene.13460, PMID: 28906581

[13] Bouslama M Barreira CM Haussen DC Rodrigues GM Pisani L Frankel MR. Endovascular reperfusion outcomes in patients with a stroke and low aspects is highly dependent on baseline infarct volumes. J Neuro Intervention Surg. (2022) 14:117–21. doi: 10.1136/neurintsurg-2020-017184, PMID: 33722970

[14] Huang L Liu Q Lu X Liu S Cao C Wang Z. Impact of encephalomalacia and white matter hyperintensities on aspects in patients with acute ischemic stroke: comparison of automated and radiologist-derived scores. Am J Roentgenol. (2022) 218:878–87. doi: 10.2214/AJR.21.26819, PMID: 34910537

[15] Jahan R Saver JL Schwamm LH Fonarow GC Liang L Matsouaka RA. Association between time to treatment with endovascular reperfusion therapy and outcomes in patients with acute ischemic stroke treated in clinical practice. JAMA. (2019) 322:252–63. doi: 10.1001/jama.2019.8286, PMID: 31310296 PMC6635908

[16] Uchida K Shindo S Yoshimura S Toyoda K Sakai N Yamagami H. Association between Alberta stroke program early computed tomography score and efficacy and safety outcomes with endovascular therapy in patients with stroke from large-vessel occlusion. JAMA Neurol. (2022) 79:1260–6. doi: 10.1001/jamaneurol.2022.3285, PMID: 36215044 PMC9552045

[17] Li L Cheng P Zhang J Wang G Hu T Sun F. Clinical effect and prognostic factors of mechanical thrombectomy in the treatment of acute ischemic stroke. Pak J Med Sci. (2022) 38:1107–12. doi: 10.12669/pjms.38.5.5723, PMID: 35799726 PMC9247756

[18] Cheng Z Geng X Rajah GB Gao J Ma L Li F. NIHSS consciousness score combined with aspects is a favorable predictor of functional outcome post endovascular recanalization in stroke patients. Aging Dis. (2021) 12:415–24. doi: 10.14336/AD.2020.0709, PMID: 33815874 PMC7990364

[19] Bourcier R Goyal M Liebeskind DS Muir KW Desal H Siddiqui AH. Association of time from stroke onset to groin puncture with quality of reperfusion after mechanical thrombectomy. JAMA Neurol. (2019) 76:405–11. doi: 10.1001/jamaneurol.2018.4510, PMID: 30667465 PMC6459219

[20] Ota T Nishiyama Y Koizumi S Saito T Ueda M Saito N. Impact of onset-to-groin puncture time within three hours on functional outcomes in mechanical thrombectomy for acute large-vessel occlusion. Interv Neuroradiol. (2018) 24:162–7. doi: 10.1177/1591019917747247, PMID: 29237321 PMC5847012

[21] Sarraj A Kleinig TJ Hassan AE Portela PC Ortega-Gutierrez S Abraham MG. Association of endovascular thrombectomy vs medical management with functional and safety outcomes in patients treated beyond 24 hours of last known well. JAMA Neurol. (2023) 80:172–82. doi: 10.1001/jamaneurol.2022.4714, PMID: 36574257 PMC9857518

[22] Rosso C Blanc R Ly J Samson Y Lehéricy S Gory B. Impact of infarct location on functional outcome following endovascular therapy for stroke. J Neurol Neurosurg Psychiatry. (2019) 90:313–9. doi: 10.1136/jnnp-2018-318869, PMID: 30425161

[23] Haranhalli N Mbabuike N Grewal SS Hasan TF Heckman MG Freeman WD. Topographic correlation of infarct area on ct perfusion with functional outcome in acute ischemic stroke. J Neurosurg. (2020) 132:33–41. doi: 10.3171/2018.8.JNS181095 30641833

[24] Panni P Michelozzi C Blanc R Chen B Consoli A Mazighi M. The role of infarct location in patients with dwi-aspects 0–5 acute stroke treated with thrombectomy. Neurology. (2020) 95:e3344–54. doi: 10.1212/WNL.0000000000011096, PMID: 33093226

[25] Seyedsaadat SM Neuhaus AA Pederson JM Brinjikji W Rabinstein AA Kallmes DF. Location-specific aspects paradigm in acute ischemic stroke: a systematic review and meta-analysis. Am J Neuroradiol. (2020) 41:2020–6. doi: 10.3174/ajnr.A6847, PMID: 33060102 PMC7658843

[26] Jang SH Lee SJ. Corticoreticular tract in the human brain: a mini review. Front Neurol. (2019) 10:1188. doi: 10.3389/fneur.2019.01188, PMID: 31803130 PMC6868423

